# Cell-in-cell phenomenon: leukocyte engulfment by non-tumorigenic cells and cancer cell lines

**DOI:** 10.1186/s12860-021-00377-3

**Published:** 2021-07-31

**Authors:** Mareike F. Bauer, Michael Hader, Markus Hecht, Maike Büttner-Herold, Rainer Fietkau, Luitpold V. R. Distel

**Affiliations:** 1 Department of Radiation Oncology, Universitätsklinikum Erlangen, Friedrich-Alexander-Universität Erlangen-Nürnberg (FAU), Universitätsstraße 27, D-91054 Erlangen, Germany; 2 Department of Nephropathology, Institute of Pathology, Universitätsklinikum Erlangen, Friedrich-Alexander-Universität Erlangen-Nürnberg (FAU), Krankenhausstraße 8-10, 91054 Erlangen, Germany

**Keywords:** Cell-in-cell, Non-professional phagocytosis, Cannibalism, Leukocyte engulfment

## Abstract

**Background:**

Research on cell-in-cell (CIC) phenomena, including entosis, emperipolesis and cannibalism, and their biological implications has increased in recent years. Homotypic and heterotypic engulfment of various target cells by numerous types of host cells has been studied in vitro and in tissue sections. This work has identified proteins involved in the mechanism and uncovered evidence for CIC as a potential histopathologic predictive and prognostic marker in cancer. Our experimental study focused on non-professional phagocytosis of leukocytes.

**Results:**

We studied the engulfment of peripheral blood mononuclear cells isolated from healthy donors by counting CIC structures. Two non-tumorigenic cell lines (BEAS-2B, SBLF-9) and two tumour cell lines (BxPC3, ICNI) served as host cells. Immune cells were live-stained and either directly co-incubated or treated with irradiation or with conventional or microwave hyperthermia. Prior to co-incubation, we determined leukocyte viability for each batch via Annexin V-FITC/propidium iodide staining.

All host cells engulfed their targets, with uptake rates ranging from 1.0% ± 0.5% in BxPC3 to 8.1% ± 5.0% in BEAS-2B. Engulfment rates of the cancer cell lines BxPC3 and ICNI (1.6% ± 0.2%) were similar to those of the primary fibroblasts SBLF-9 (1.4% ± 0.2%). We found a significant negative correlation between leukocyte viability and cell-in-cell formation rates. The engulfment rate rose when we increased the dose of radiotherapy and prolonged the impact time. Further, microwave hyperthermia induced higher leukocyte uptake than conventional hyperthermia.

Using fluorescent immunocytochemistry to descriptively study the proteins involved, we detected ring-like formations of diverse proteins around the leukocytes, consisting, among others, of α-tubulin, integrin, myosin, F-actin, and vinculin. These results suggest the involvement of actomyosin contraction, cell-cell adhesion, and the α-tubulin cytoskeleton in the engulfment process.

**Conclusions:**

Both non-tumorigenic and cancer cells can form heterotypic CIC structures by engulfing leukocytes. Decreased viability and changes caused by microwave and X-ray irradiation trigger non-professional phagocytosis.

**Supplementary Information:**

The online version contains supplementary material available at 10.1186/s12860-021-00377-3.

## Background

In recent years, a field of science has grown up around a phenomenon long known of, but previously largely overlooked: cell-in-cell (CIC) structures. The term refers to a phenomenon whereby one cell is inside another [1] as a result of non-professional phagocytosis [2]. Cell-in-cell structures can arise from the interactions of cells of the same cell type (homotypic CIC) or different cell types (heterotypic CIC). Entosis, emperipolesis and cannibalism are three manifestations of this phenomenon, each of which features differences in the formation mechanisms and biological impact of cell-in-cell structures [1].

Entosis is the homotypic active invasion of one cell into another. In addition to cell-cell adhesion, entosis requires a contractile force [3] and a connecting sensor to trigger the uptake [4]. The engulfment of living leukocytes by other cells is called emperipolesis [5]. Cannibalism is defined as the ability of a cancer cell to engulf living or dead cells or even amorphous material [1]. A complex set of factors, pertaining both to the host cell and to the engulfed cell, regulates all three of these phenomena [6]. Actomyosin cytoskeleton rearrangements, cell-cell adhesion and a mechanosensitive interfacing ring [4] are some of the key players in non-professional phagocytosis [1, 4, 7, 8].

Cell-in-cell structures are part of physiological processes, such as cell maturation [7, 9] tissue development [10] and homeostasis [11], and also occur in pathological processes, namely inflammation [12] and tumour formation [9, 11, 13]. Identifying and understanding the biological effects of cell-in-cell structures in cancer has become a focal area of research.

Non-professional phagocytosis can generate distinctly divergent, indeed opposing effects in the emergence of tumours. It can support tumour development and progression, serving to supply nutrients and giving the host a survival advantage [1, 6, 14, 15]. The invasion of one cell into another can lead to multinucleation, promoting aneuploidy and malignant degeneration [11, 13]. The formation of cell-in-cell structures also acts as a selection mechanism for the most malignant clones, as they are more potent phagocytes than are less malignant clones [16]. Further, tumour cells establish an immune escape mechanism by engulfing targeting immune cells [11, 15, 17]. This alters the tumour microenvironment [13]. However, non-professional phagocytosis also fulfils a tumour-suppressive role. It can clear aberrant cells from tissues and thereby prevent aneuploidy and cancerous degeneration [8, 18]. It can also prevent the formation of metastases by clearance of matrix-detached cells [15]. Additionally, incorporated immune cells can cause host cell death through cytotoxic effects released inside the host cell [9, 19].

Notwithstanding this ambiguity in the biological impact of cell-in-cell structures, Fais and Overholtzer have declared them a “hallmark of cancer” [1]. Studies on cell-in-cell structures in various tumours point to CIC as a potential histopathologic predictive or prognostic marker for cancer [20–27].

Against this backdrop, our experimental study aimed to shed light on the role of leukocytes in non-professional phagocytosis. Due to the assertion that non-professional phagocytosis is a characteristic of malignant clones [1, 13, 14, 28], we focussed on leukocyte interaction with both non-tumorigenic and tumour tissue cells in vitro to determine differences between malignant and non-malignant cells. In addition, our study sought to establish whether engulfment differs in accordance with the viability of target cells. This study uses the term “leukocytes” as a synonym for isolated peripheral blood mononuclear cells (PBMC).

## Results

### Non-tumorigenic tissue and cancer cell lines have the capacity to engulf leukocytes

Recent experiments run in our laboratory found that dead target cells resulted in a higher rate of homotypic non-professional phagocytosis [29]. Assuming heterotypic non-professional phagocytosis has similar properties, we initially used leukocytes exposed to 56 °C hyperthermia as target cells.

**Fig. 1:**
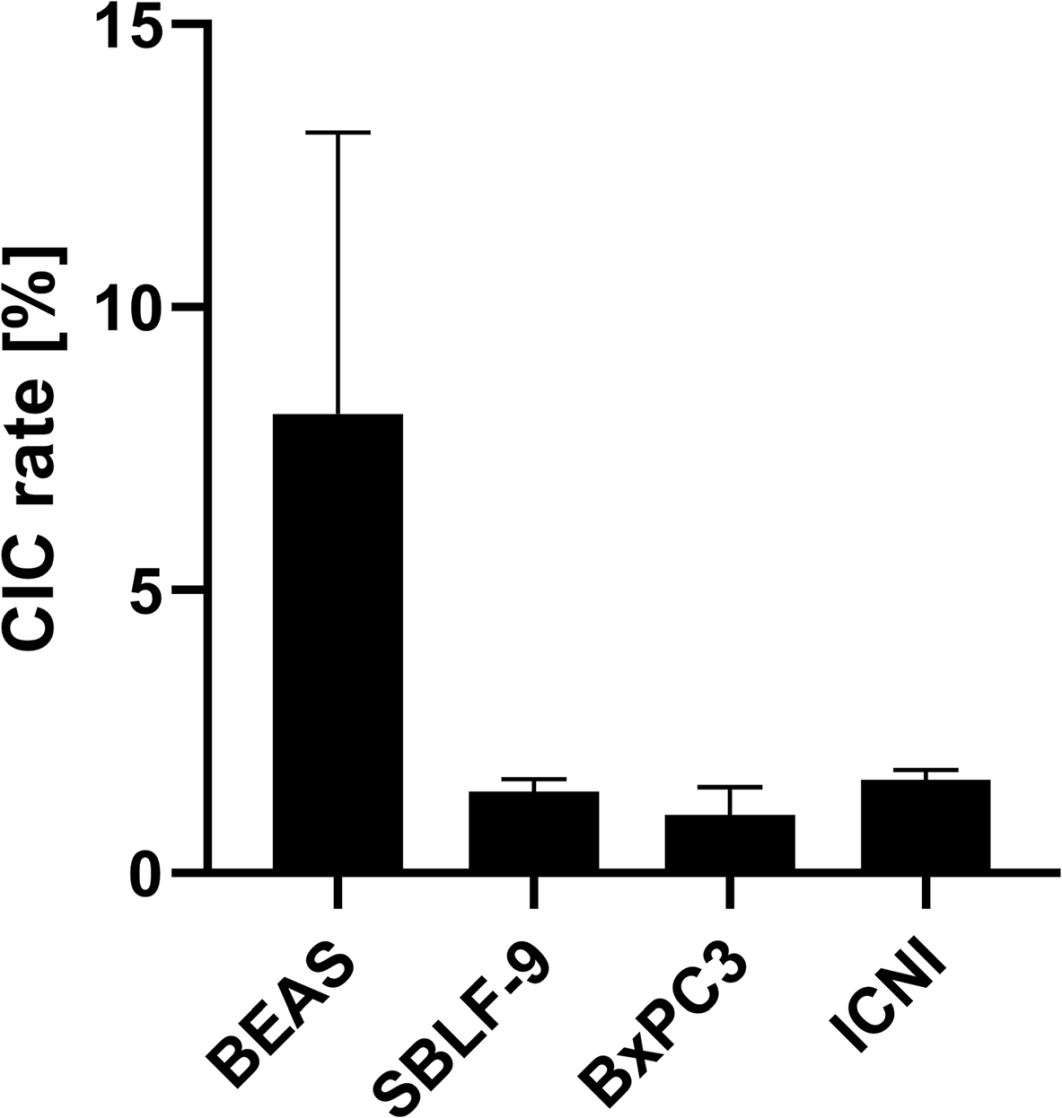
Non-professional uptake of leukocytes into different host cells lines. Heated (56 °C, 40 min) leukocytes are co-incubated for 4 h with adherent epithelial lung cells (BEAS-2B), primary human fibroblasts (SBLF-9), pancreatic cancer cells (BxPC3) and melanoma cells (ICNI). The leukocytes have been isolated from whole blood residues from platelet donations and from EDTA tubes from healthy blood donors. A Kruskal-Wallis test detects a significant difference between the phagocytic capacities of BEAS-2B and BxPC3 (*p* = 0.005)

All recipient cell lines studied were able to engulf hyperthermia-treated leukocytes. However, they showed differing capacities for engulfment. CIC rates ranged from 8.1% ± 5.0% in the immortalised human lung epithelial cell line BEAS-2B to 1.0% ± 0.5% in the human primary pancreatic adenocarcinoma cell line BxPC3. The engulfment capacity of BEAS-2B was significantly higher than the capacity of BxPC3 (*p* = 0.005). The cancer cell lines BxPC3 and ICNI (1.6% ± 0.2%) engulfed the leukocytes at a similar rate as did the human primary fibroblasts SBLF-9 (1.4% ± 0.2%) (Fig. 1 and Table 1).

**Table 1:** Heterotypic engulfment capacity of each cell line

	BEAS-2B	SBLF-9	BxPC3	ICNI
Mean	8.1%	1.4%	1.0%	1.6%
Standard deviation	5.0%	0.2%	0.5%	0.2%

As we had obtained low CIC rates for SBLF-9, BxPC3 and ICNI, we decided to perform further experiments with BEAS-2B as host cells. We hoped these experiments would enable us to determine differences between treatments applied previous to co-incubation.

### Inverse correlation between CIC rate and leukocyte viability

**Fig. 2:**
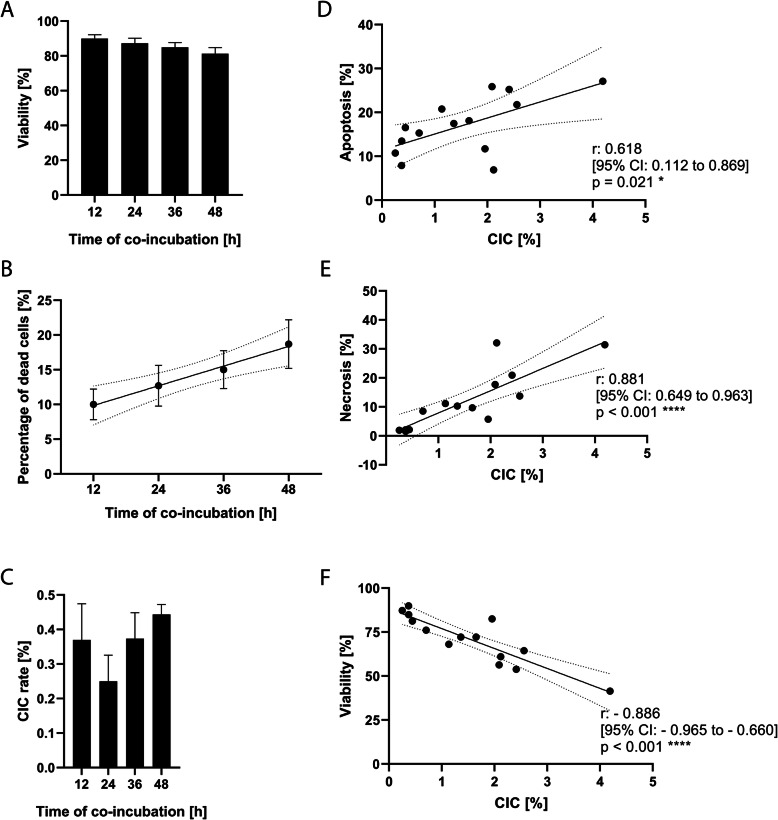
Cell-in-cell (CIC) rates are positively associated with apoptosis and necrosis and negatively with viability. **A** Viability of live-stained peripheral blood mononuclear cells (PBMC) co-incubated for 12 h to 48 h with BEAS-2B as host cells. **B** Linear regression of the death rate of PBMC over co-incubation time. **C** CIC rate over co-incubation time. Correlation of (**D**) apoptosis and (**E**) necrosis rates and (**F**) viability with CIC rates

Living leukocytes were co-incubated with BEAS-2B for 12 h, 24 h, 36 h or 48 h. The viability of the leukocytes declined from 90.0% ± 2.2% at 12 h to 81.3% ± 3.5% at 48 h (Fig. 2A), which implies that the vital leukocytes died over time at a certain rate (Fig. 2B). However, the CIC rate varied only marginally over time (Fig. 2C).

Overall, we found significant positive correlations between CIC rates and both apoptotic (*p* = 0.021, Fig. 2D) and necrotic rates (*p* < 0.001, Fig. 2E), as well as a significant negative correlation between CIC rates and viability (*p* < 0.001, Fig. 2F). It appears, therefore, that dying and dead leukocytes are more susceptible to targeting by non-professional phagocytosis.

### CIC rate depends on irradiation dose and impact time

**Fig. 3:**
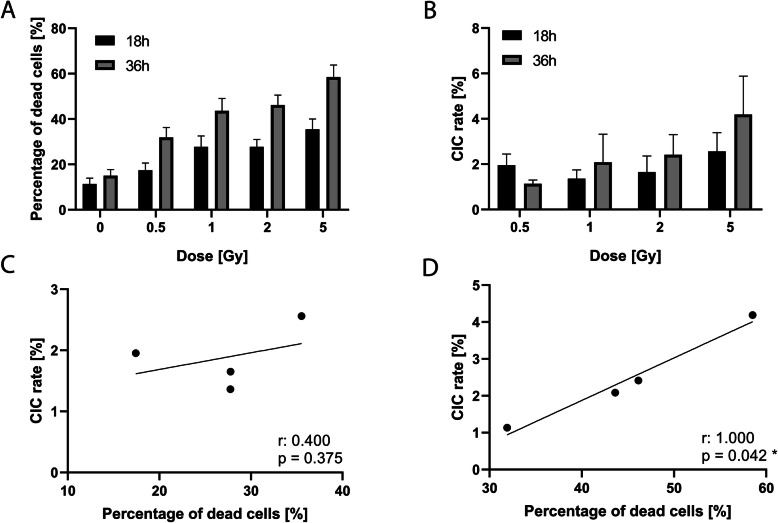
Effect of irradiation and its impact time on leukocyte uptake. Leukocytes are irradiated and incubated for 18 h or 36 h to allow radiation damage to occur. They are then co-incubated for 4 h with BEAS-2B as host cells. Untreated leukocytes serve as negative controls. **A** Proportion of dead cells by dose and time. **B** Cell-in-cell (CIC) rates by dose and time. Correlation of death rates and CIC rates after impact times of **C** 18 h and (**D**) 36 h

**Fig. 4:**
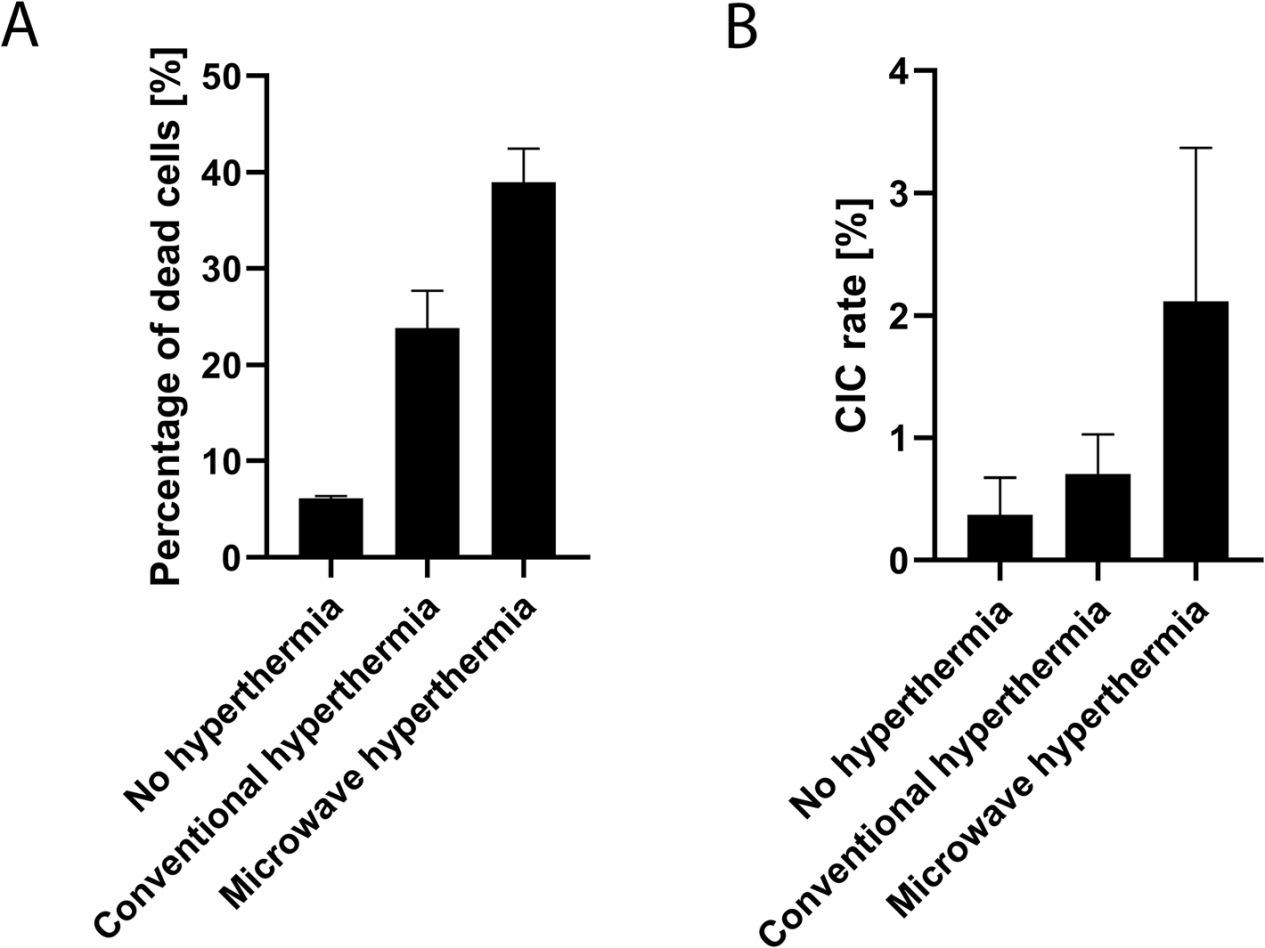
Comparison of conventional and microwave hyperthermia. Leukocytes are exposed to conventional hyperthermia via water bath or microwave hyperthermia at 44 °C for 1 h. Leukocytes not exposed to hyperthermia are the negative control. **A** Cell death rates and **B** CIC rates are determined after 4 h of co-incubation

We tested various treatments prior to co-incubation in order to determine whether the cause of cell death impacts engulfment. The leukocytes were irradiated with 0.5Gy, 1Gy, 2Gy or 5Gy, then incubated for 18 h and 36 h to ensure that the damage caused by irradiation could progress prior to co-incubation [30, 31]. The viability of untreated leukocytes after 18 h or 36 h respectively served as negative controls. As the engulfment rate of untreated leukocytes remained mostly constant from 4 h (neg. Control Fig. 4A) to 48 h (Fig. 2C), we did not perform additional negative control experiments for CIC rates at 18 h. With an increasing dose of irradiation, the leukocytes’ viability rate decreased, while apoptotic and necrotic rates increased. Death rates also rose with latency, as cells had more time to undergo apoptosis as a response to irradiation damage (Fig. 3A). After an impact time of 18 h, the CIC rate varied only marginally, whereas it increased in line with irradiation dose after an impact time of 36 h (Fig. 3B). This means that no correlation between CIC rate and death rate was observed after an impact time of 18 h (*p* = 0.375, Fig. 3C), but after an impact time of 36 h, a significant positive correlation between CIC rate and death rate emerged (*p* = 0.042, Fig. 3D).

### Microwave irradiation increases both death rate and CIC rate

A number of research groups have found non-thermal effects of microwave irradiation on cells [32–34]. We investigated whether these effects also manifested in non-professional phagocytosis of leukocytes. The leukocytes were exposed to 44 °C, generated either by microwave irradiation or water bath, for a period of 1 h. Viability and CIC rates after 4 h of co-incubation were compared. Viability and CIC rates of untreated leukocytes at the same points in time served as negative controls. Overall death rates induced by conventional hyperthermia and microwave hyperthermia did not differ significantly (Fig. 4A). However, differences did appear between apoptosis and necrosis rates. Conventional hyperthermia resulted in around twice as much apoptosis as necrosis. By contrast, microwave irradiation led to nearly 5 times more necrosis than apoptosis. The CIC rates were 2.12% ± 1.26% after microwave irradiation and 0.70% ± 0.33% after treatment with conventional hyperthermia (Fig. 4B). This difference was not significant.

### Various proteins form a ring-like structure around the leukocyte during and after engulfment

**Fig. 5:**
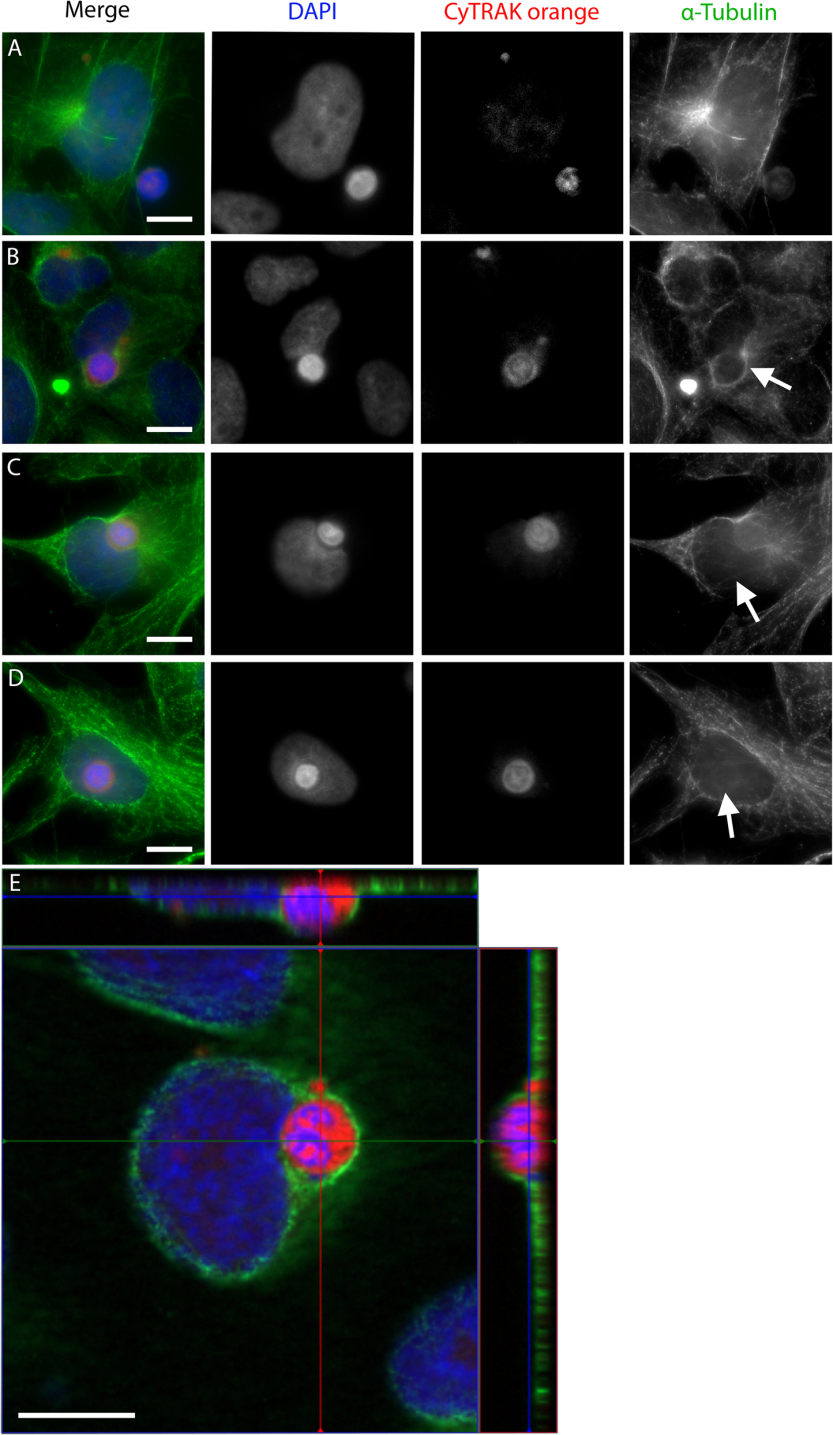
Role of α-tubulin in leukocyte uptake. α-Tubulin is stained using a specific primary antibody and Alexa Fluor 488 secondary antibody. CyTRAK orange is the live dye used to stain peripheral blood mononuclear cells before co-incubation with BEAS-2B as host cells. The nuclei are stained with DAPI. The figure displays several stages of engulfment of leukocytes by BEAS-2B, as a representative sample of > 150 CIC structures analysed: **A** α-tubulin cytoskeleton of a BEAS-2B host cell and a leukocyte before internalisation. **B** Establishment of a link between target and host cell; arrow marks ring-like condensation around leukocyte. **C** Host cell nucleus deformation; arrow demonstrates connection between α-tubulin ring and cytoskeleton of recipient cell. **D** Complete engulfment of the leukocyte by a BEAS-2B cell; arrow indicates ring-like structure still present around engulfed leukocyte. **E** Merged X-Y-Z scan of a representative cell-in-cell structure shows complete engulfment of leukocyte. Scale bars indicate 10 μm

Detection of proteins involved in cell-in-cell formation proceeded via immunofluorescence microscopy. We used PBMC as target cells and BEAS-2B as host cells, and analysed cytoskeletal proteins and proteins involved in cell-cell adhesion. In α-tubulin staining, a ring formed around the leukocyte during every stage of the uptake (Fig. 5B). This ring was connected to extensions of the α-tubulin cytoskeleton of the engulfing cell (Fig. 5C). The internalising cell seemed to pull in the leukocyte from the opposite pole of the nucleus. The ring-like α-tubulin condensation around the leukocyte remained visible after engulfment was complete (Fig. 5D). An X-Y-Z scan image illustrates the complete engulfment of a leukocyte by an α-tubulin-stained BEAS-2B cell (Fig. 5E).

**Fig. 6:**
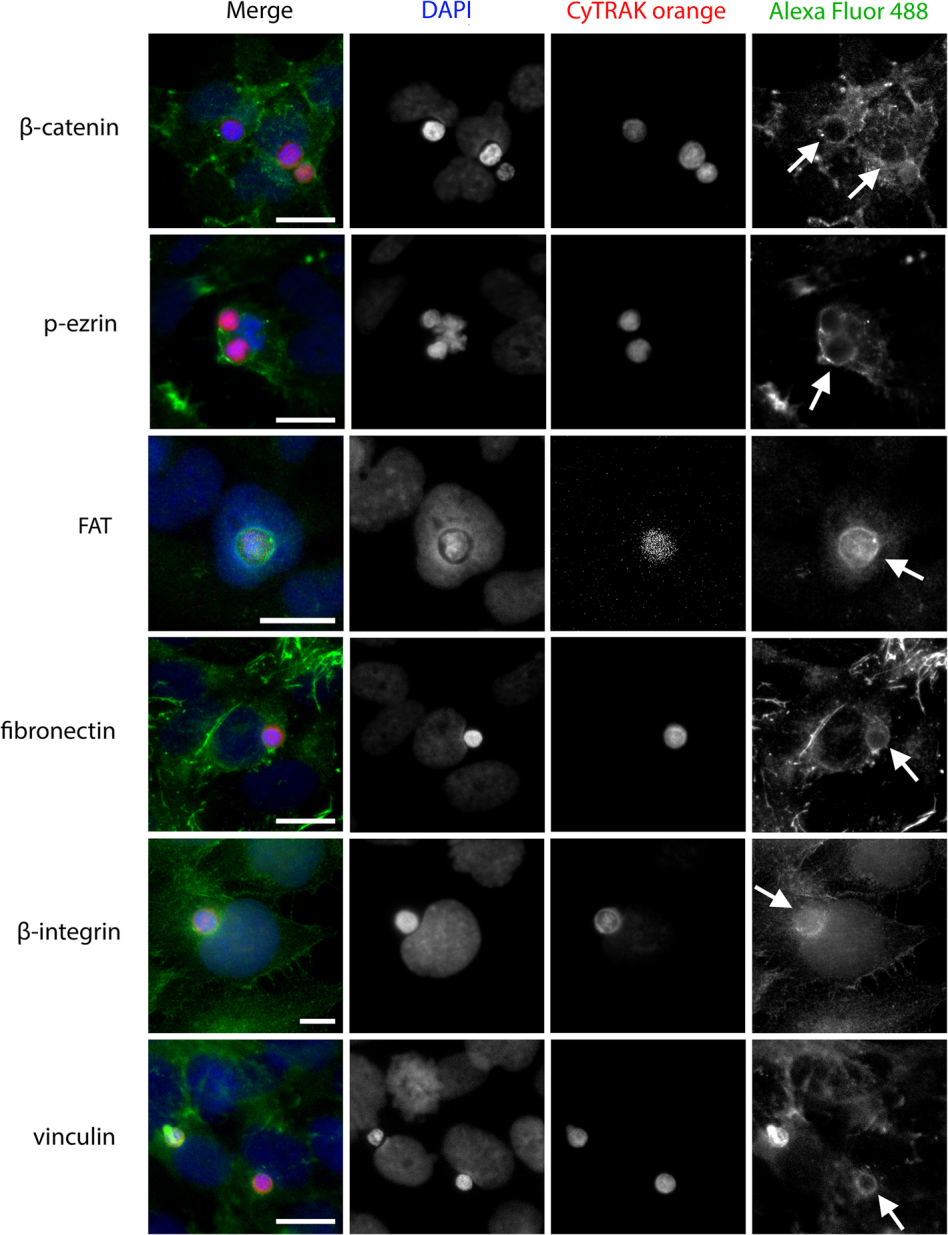
Ring-like structures surrounding ingested leukocytes are formed by many different proteins. All images result from co-incubation of peripheral blood mononuclear cells with BEAS-2B as host cells for 4 h. They are representative of > 150 CIC structures analysed. The images depict β-catenin, p-ezrin, FAT, fibronectin, β-integrin and vinculin staining. The proteins are targeted by specific primary antibodies and all stained with Alexa Fluor 488 secondary antibodies. Leukocytes were live stained with CyTRAK orange before co-incubation. DAPI is used to stain the nuclei. Scale bars indicate 10 μm. Arrows indicate ring-like structures

Further, β-integrin formed a ring around the leukocyte during engulfment, with agglomeration at the contact side (Fig. 6). Similar ring-like protein condensations were observed in β-catenin, p-ezrin, FAT, fibronectin and vinculin staining (Fig. 6).

**Fig. 7:**
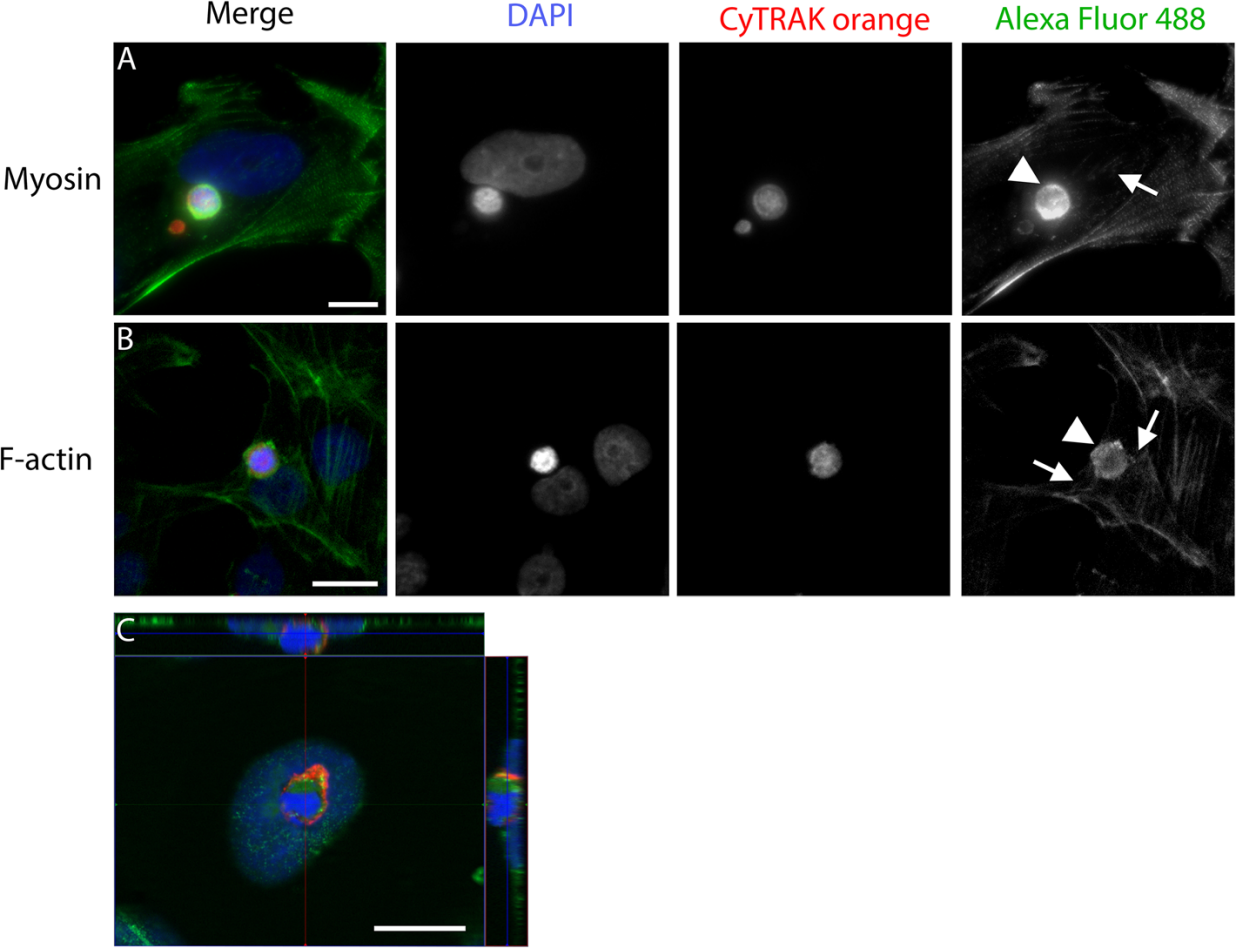
Actomyosin distribution during non-professional phagocytosis. Peripheral blood mononuclear cells are stained with CyTRAK orange before co-incubation with BEAS-2B for 4 h. Nuclei are stained using DAPI. **A** Myosin staining is performed using a secondary antibody coupled with Alexa Fluor 488. **B** Actin is visualised using phalloidin. The images shown here are representative of > 150 CIC structures analysed. **C** Merged X-Y-Z scan of myosin-stained cell-in-cell structure to demonstrate the complete engulfment of the leukocyte. Scale bars indicate 10 μm. Arrowheads indicate ring-like structures; arrows indicate interactions with the cytoskeleton of the engulfing cell

As the F-actin cytoskeleton and myosin cytoskeleton are key players in professional phagocytosis, we also studied these proteins (Fig. 7). Both formed a ring-like structure around the leukocyte, like the ones described above. Further, a slight connection to the actomyosin cytoskeleton of the outer cell was occasionally visible. However, we did not observe the pseudopods of the actin cytoskeleton that are typical of professional phagocytosis.

## Discussion

The cancer cell lines used in this study formed CIC structures by engulfing leukocytes, thus exhibiting behaviour consistent with that found by other authors [14, 17, 19, 26, 27, 35]. The non-tumorigenic cell lines SBLF-9 and BEAS-2B also engulfed leukocytes under normal cell culture conditions. In line with the findings of previous work [14, 28], primary human melanoma cells did not exhibit high phagocytic potential in this study. Their CIC rate did not differ from the CIC rate of human primary skin fibroblasts. It therefore appears that malignant transformation alone is insufficient to increase the phagocytic capacity of skin cells. In accordance with the findings of He et al. [36], we assume that differences in host tumour cell biology play a role in leukocyte-tumour cell interaction and may explain the observed differences in phagocytic capacities.

As the BEAS-2B host cells engulfed live as well as apoptotic and necrotic leukocytes, our experimental setting most likely resembles the phenomenon called “cannibalism”. In a similar way to the BEAS-2B host cells, cannibalistic cancer cells do not discriminate between living and dead cells and even engulf amorphous material [1, 28, 37]; this notwithstanding, in our experiments dead cells were more likely to be targets of engulfment. As the dead cells lay motionless on the host cell layers, they may have sunk into the host cells, in a manner similar to the description of cancer cell cannibalism by Stefano Fais [37]. The fact that living cells retain their movement may explain the higher engulfment rates for dead cells.

Besides a decrease in viability, the CIC rate also depended on other factors. After X-ray and microwave irradiation, leukocyte engulfment rates were comparable at similar death rates. Untreated leukocytes and those exposed to hyperthermia in a water bath were much less frequently engulfed than irradiated leukocytes at similar death rates. It appears, then, that non-thermal effects of microwave irradiation [32–34] and cellular effects and changes caused by X-ray irradiation also trigger leukocyte engulfment.

Although the mechanism behind our experimental setting predominantly resembles cannibalism, the changes we observed at protein level also show parallels to entosis. In what follows, we will therefore review our findings in the light of the various mechanisms of uptake described in the literature.

As shown by Xia et al., microtubules are important for entosis and disturbing them will disrupt cell-in-cell formation [38]. In the present study, we also observed structural changes of α-tubulin during cell-in-cell formation. The α-tubulin cytoskeleton deformed around the leukocyte or appeared to “pull it in”. Our findings therefore indicate involvement of the microtubule cytoskeleton in the process of non-professional phagocytosis.

Alongside its role in cellular plasticity, the microtubule cytoskeleton affects the stiffness of the cell, as does the actomyosin cytoskeleton [16, 38–40]. Actomyosin is concentrated inside the internalised cell, particularly opposite the adhesion contacts, and represents the contractile force responsible for invasion of the host cell [16, 39]. Fluorescent immunocytochemistry has consistently revealed actomyosin enhancement at the cell membrane of the leukocyte. As a large number of leukocytes in our experiments were necrotic, we assume that an active invasion process, as occurs in entosis [3], is improbable here. This notwithstanding, the stiffness of the leukocytes may be increased, possibly by passive myosin enhancement, which may have precipitated engulfment of that leukocyte.

Research has proposed that adherens junctions act as sensors of cellular stiffness in cell-in-cell formation, thereby mediating entosis [8]. Vinculin is one of the linkers between adherens junctions and the actomyosin cytoskeleton [4, 8]. In our experiments, vinculin was condensed around the internalised leukocyte in a ring-like structure. This ring may be a two-dimensional representation of the mechanosensitive formation of vinculin with other proteins described by Wang et al., which is required for leukocyte engulfment [4]. One of the proteins involved could be β-catenin, as it was distributed in a similar manner to vinculin both in Wang et al. [4] and in our experiments. However, the ring-like β-catenin formation around the leukocytes could also indicate that the leukocytes reside inside a vacuole formed by the cell membrane of the engulfing cells [19].

Various integrins mediate cell-cell adhesions, for example in leukocyte transcytosis [41, 42], or in epithelial to mesenchymal transition, a prerequisite for cancer cell invasion and metastasis formation [43]. Sexton et al. demonstrated that integrins participate in the detection of apoptotic leukocytes, which leads to their engulfment [44]. They also described an integrin enhancement around the target cell [44]. In our experiments, β-integrin was condensed at the contact sides between the target and host cell at an early stage of the engulfment. Once the leukocyte was completely engulfed, the β-integrin formed a ring-like structure around it. This may indicate the formation of focal adhesions [43, 44] prior to engulfment as means of cell-cell contact.

Fibronectin is one of the proteins that interact with integrins [45]. It is involved in cell-matrix attachment, cell-cell adhesion [45, 46] and phagocytosis, especially of bacteria [47]. In our experiments, fibronectin formed ring-like structures around the leukocytes, suggesting it could be displayed at their surface [46]. This display may have been triggered by hyperthermia treatment or similar stress factors, resulting in a form of leukocyte activation [46] prior to cell death. This could mediate non-professional phagocytosis via interaction with host cell integrins.

The actin cytoskeleton’s linker protein, ezrin, also takes part in non-professional phagocytosis [28, 35]. It is distributed around the internalised particle [28]; in our case it formed a ring-like structure around the leukocytes. The inhibition of ezrin greatly impairs the phagocytic capacity of tumour cells [28, 35]. Our findings suggest that ezrin is part of the regulatory mechanisms of non-professional phagocytosis by non-tumorigenic cells. We would recommend that future studies in this area focus on differences in ezrin levels and ezrin’s phosphorylation in cells with high versus low phagocytic capacity, as phosphorylation patterns may potentially explain the disparity [28].

Another protein involved in the regulation of actin cytoskeleton transitions is FAT1 [48]. This is a transmembrane protein from the cadherin superfamily, particularly expressed in epithelial cell lines. FAT1 has been shown to mediate cell-cell adhesion [48]. Further, it can bind to β-catenin [49]. This may explain why we found a similar distribution of FAT1 and β-catenin. The exact role of FAT1 in the interplay of cytoskeleton rearrangement and cell-cell adhesion remains to be identified.

## Conclusion

This study’s findings are indicative of the ability of non-tumorigenic and cancer cells to engulf leukocytes and form heterotypic cell-in-cell structures. They also suggest that the phagocytic potential of a cell line does not solely depend on its malignant transformation, but additionally on a complex set of biological factors not analysed here. In our experiments, decreased viability and exposure to X-ray and microwave irradiation increased rates of non-professional phagocytosis.

## Methods

### Cell culture

We studied the phagocytic capacity of two non-tumorigenic and two cancer cell lines. As non-tumorigenic cell lines, we used BEAS-2B, a virus-transformed epithelial lung cell line, and SBLF-9, a primary skin fibroblast cell line. The cancer cell lines chosen were BxPC3, a human pancreatic adenocarcinoma cell line, and ICNI, a human melanoma cell line. Other studies conducted in our laboratory had found BEAS-2B, several primary skin fibroblast cell lines and BxPC3 to display homotypic non-professional phagocytosis [29, 50]. BxPC3 and a primary skin fibroblast cell line also engulfed heterotypic cells [50]. In addition to this, the work of He et al. demonstrated the ability of BxPC3 to engulf leukocytes [36]. On this basis, we expected these three cell lines to be most likely to engulf leukocytes. Lugini et al. demonstrated the ability of metastatic melanoma cells to engulf leukocytes both in vivo and in vitro [14]. In light of this finding, we included a human melanoma cell line in our study. Our aim in including both primary human fibroblast and melanoma cell lines was to directly compare the uptake capacity of healthy tissue to that of tumour tissue.

Host cells were cultured adherently on glass coverslips in 6-well plates at 37 °C in a 5% CO_2_ atmosphere, reaching a confluent layer of approximately 200,000 cells at the time of co-incubation. We used individually composed media, comprising different amounts of F-12 Medium, Dulbecco’s Modified Eagle’s Medium, foetal calf serum (FCS) and variable additives, including 1% penicillin/streptomycin antibiotics.

### Isolation of leukocytes from peripheral blood

Peripheral blood mononuclear cells (PBMC) were used as target cells. They were isolated both from whole blood residues from platelet donations and from EDTA (ethylenediaminetetra-acetic acid) tubes from healthy blood donors. The Division of Transfusion Medicine at Erlangen’s university hospital (Universitätsklinikum Erlangen) provided the blood residues. The blood was transferred from its collection tubes to a 50 ml centrifuge tube and mixed with phosphate-buffered saline (PBS) to produce equal volumes of 50 ml. Fresh 50 ml centrifuge tubes were filled with 15 ml of Lymphoflot (BioRad, Feldkirchen, Germany) each, and we carefully superimposed 12.5 ml of the mixture of blood and PBS on the Lymphoflot. The tubes were centrifuged for 20 min at 850 g at room temperature with a low acceleration and with no brake. This process separated the various cell fractions of the blood samples due to the density gradient of Lymphoflot. The rings containing PBMC were transferred to fresh 50 ml centrifuge tubes and mixed with cold PBS-EDTA. The tubes were centrifuged again for 12 min at 4 °C and 300 g. The resulting PBMC pellet was resuspended in PBS-EDTA and centrifuged for 12 min at 4 °C and 200 g. After these washing steps, we resuspended the PBMC pellet in 50 ml of RPMI Medium with 1% penicillin/streptomycin antibiotics and 10% FCS (R-10 Medium) (all from Gibco, Schwerte, Germany). The concentration of PBMC was calculated using a Neubauer counting chamber.

### Non-professional phagocytosis assay in vitro

In previous research, our working group had established a protocol for the study of non-professional phagocytosis of necrotic and apoptotic cells in vitro [50]. We slightly modified this protocol for these experiments to take account of particular characteristics of leukocytes.

For all experiments, PBMC were stained using live dye, CyTRAK orange (Thermo Fisher, Schwerte, Germany). The excess dye was washed off and the target cells were resuspended in R-10 Medium. After staining, PBMC were exposed to hyperthermia at 56 °C for 40 min in a water bath. Before co-incubation, adherent host cell layers were washed with medium to remove cell debris. The medium was replaced. Stained and hyperthermia-treated target cells were added to the fresh medium. The 6-well plates were shaken carefully to distribute the target cells evenly on the cover slips. We co-incubated host cells with target cells at 37 °C in a 5% CO_2_ atmosphere.

Initially, we co-incubated the cells for 4 h and used a 1:1 ratio of target and host cells. After finding a low rate of engulfment of stained, untreated leukocytes after 4 h of co-incubation, we tested longer co-incubation times (12 h, 48 h, 36 h, 48 h). We also increased the target-to-host cell ratio to 2:1 by adding 400,000 target cells to an adherent layer of 200,000 BEAS-2B as host cells. In these experiments, viability and death rates were determined after co-incubation, using stained, otherwise untreated leukocytes.

### Variation of treatment prior to co-incubation

To determine if the cause of cell death impacted the uptake of leukocytes, we varied the treatment applied prior to co-incubation. PBMC were used as target cells and stained with CyTRAK orange. Due to the comparably high phagocytic capacity they had shown in our standard non-professional phagocytosis assay, we chose BEAS-2B as host cells for these experiments. To potentially increase CIC rates and better determine possible differences between the treatments applied prior to co-incubation, we opted for a target-to-host cell ratio of 2:1.

One batch of live stained PBMC was irradiated with 0.5Gy, 1Gy, 2Gy or 5Gy and then incubated for 18 h or 36 h at 37 °C and 5% CO_2_ to allow radiation damage to occur. We determined their viability and co-incubated them for 4 h with the recipient cells. A further batch of live stained PBMC was exposed to hyperthermia at 44 °C for 1 h. We compared conventional thermal hyperthermia generated by a water bath to microwave hyperthermia. The system for delivering microwave irradiation at 2.45 GHz, as described by Hader et al., enables hyperthermia under controlled conditions in a sterile environment [51]. After exposure to hyperthermia, the PBMC were co-incubated with the recipient cells for 4 h. After this, we determined the viability of the PBMC.

### Fluorescent immunocytochemistry

For further staining, the cells were permeabilised and fixed at room temperature with a solution containing 3.7% formaldehyde and 0.1% Triton X-100. We washed the samples with PBS three times. Then, the samples were incubated at 4 °C overnight with a blocking solution containing 5% FCS, 0.3% Triton X-100 and 0.3% sodium azide in PBS. Primary and secondary antibodies were diluted in a solution containing 0.1 g bovine serum albumin and 30 μl Triton X-100 in PBS. The samples were incubated overnight once again, this time with the primary antibody dilution in a humidity chamber at 4 °C. The next day, the samples were washed three times with PBS and then incubated with the secondary antibody dilutions for 1.5 h at room temperature in a humidity chamber. Samples were washed three times with PBS again before drying. Dry samples were mounted with Prolong Gold with DAPI (4′,6-diamidino-2-phenylindole) (Thermo Fisher, Schwerte, Germany). Supplementary Tables 1 and 2 list the primary and secondary antibodies used for the experiments. Actin was stained using phalloidin conjugated with Alexa Fluor 488 diluted 1:500 to 1:1000. The images were acquired using a fluorescence microscope (AxioImager Z2, Zeiss, Göttingen, Germany).

### Image analysis and CIC count

We used Biomas software to analyse the images. Prior to analysis, we marked CIC structures manually on the images. The software then counted the number of marked CIC structures and the number of host cells. A structure was classified as one CIC if one or more red-stained leukocytes were engulfed by one recipient cell. The process of internalisation deformed the host cell’s nucleus, while the leukocytes retained an intact nucleus and a round shape. The CIC rate calculated by the software is the quotient of the number of CIC structures and the number of host cells. CIC rates are expressed as a percentage.

### Annexin V-FITC/propidium iodide staining

For each batch, Annexin V-FITC (BioLegend, San Diego, CA, USA)/propidium iodide (Thermo Fisher, Schwerte, Germany) staining was performed to determine numbers of viable, apoptotic and necrotic leukocytes. 1 × 10^5^ cells were centrifuged and the pellet was resuspended with 0.1 μg/ml Annexin V-FITC and 0.2 μg/ml propidium iodide in Ringer’s solution. We incubated the samples for 30 min at 4 °C in the dark and subsequently analysed the samples using flow cytometry.

### Statistical data analysis

We used GraphPad Prism version 8 (GraphPad Software, San Diego, CA, USA) for data analysis and plotting. Each assay was performed at least three times. We calculated the mean and the standard deviation of the results. Apoptotic and necrotic cell rates were added together to obtain death rates. As the data did not follow a Gaussian distribution, we calculated the Spearman correlation coefficient. Kruskal-Wallis tests were performed to analyse the phagocytic capacity of the various host cells. We performed Mann-Whitney U-tests to compare the effect of different impact times after irradiation on CIC rates and death rates, depending on the dose applied. We also used Mann-Whitney U-tests to analyse the difference in CIC rates and death rates after conventional and microwave hyperthermia.

## Supplementary Information

**Additional file 1: Table S1.** Primary antibodies used for immunofluorescence staining. **Table S2.** Secondary antibodies used for immunofluorescence staining.

## Data Availability

All data generated and analysed in the current study are available from the corresponding author on reasonable request.
