# Supplementary material for: Cell-in-cell phenomenon: leukocyte engulfment by non-tumorigenic cells and cancer cell lines
Source: BMC Mol Cell Biol. 2021 Jul 31;22:39. doi: 10.1186/s12860-021-00377-3 (PMC8325834; doi:10.1186/s12860-021-00377-3)
Supplement: Supplementary file 1 — Additional file 1: Table S1. Primary antibodies used for immunofluorescence staining. Table S2. Secondary antibodies used for immunofluorescence staining. [file 12860_2021_377_MOESM1_ESM.docx]

Supplement

Supplementary table 1: Primary antibodies used for immunofluorescence staining

| **Target of antibody** | **Species** | **Dilution** | **Company name** |
| --- | --- | --- | --- |
| β-Catenin | Mouse | 1:300 | Becton Dickinson |
| p-Ezrin | Mouse | 1:100 | Becton Dickinson |
| FAT | Rabbit | 1:250 | abcam |
| Fibronectin | Rabbit | 1:200 | abcam |
| β-Integrin | Mouse | 1:250 | abcam |
| Myosin | Rabbit | 1:250 | abcam |
| α-Tubulin | Rabbit | 1:250 | abcam |
| Vinculin | Rabbit | 1:100 | abcam |

Supplementary table 2: Secondary antibodies used for immunofluorescence staining

| **Target species of antibody** | **Species** | **Dilution** | **Fluorophore** | **Company name** |
| --- | --- | --- | --- | --- |
| Mouse | Goat | 1:400 | Alexa Fluor 488 | Invitrogen |
| Rabbit | Goat | 1:200 | Alexa Fluor 488 | Invitrogen |
| Rabbit | Goat | 1:1000 | Alexa Fluor 488 | Invitrogen |
